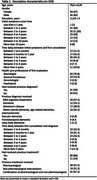# Alzheimer's Disease Diagnostic Paradox; New Biomarkers and Disease‐Modifying Therapies, but the Diagnosis is Still Delayed. A Real‐World Survey in Buenos Aires

**DOI:** 10.1002/alz70860_102209

**Published:** 2025-12-23

**Authors:** Julian Fernandez Boccazzi, Guido Santiago Dorman, Maria Belen Martin Escandell, Ignacio Flores, Noelia Pontello, Florencia Vallejos, Galeno Rojas, Santiago O'Neill

**Affiliations:** ^1^ Neuroscience Institute, Favaloro Foundation, Buenos Aires, Argentina; ^2^ Neuroscience Institute, Favaloro Foundation, Buenos Aires, Buenos Aires, Argentina

## Abstract

**Background:**

The advent of new diagnostic tools and the novel disease‐modifying treatment approval have started changing Alzheimer disease (AD) situation. However, because these drugs are approved in initial stage of the disease, it is crucial to diagnose in early stages to allow patients to be eligible to new treatments The objective of this study was to describe the time between the onset of symptoms, initial consultation, and diagnosis in patients with cognitive impairment.

**Method:**

A cross‐sectional observational study was conducted. An anonymous survey was developed for patients who consulted to a memory clinic with cognitive complains. Questions were answered by patients’ partners. We include the onset time of cognitive symptoms, the time of first consultation with any health professional, as well as the time between first consultation and diagnosis. A descriptive statistical and frequency assessment were performed.

**Result:**

To date, 104 patients have been included. Mean age was 76.6±6.2 years. A 31% experienced cognitive symptoms onset within of one to two years. 44% attended for first time to health care professional between one and two years after symptoms onset. Half of them had their first consultation at the time of completing the survey. Eighty percent reported that their initial evaluation for cognitive symptoms was performed by a neurologist. Only 13% had their first evaluation by a general practitioner. A 59% of patients had received a previous diagnosis, with a time delay from first consultation to diagnosis between six months to two years in 56% of the cases. Mild cognitive impairment was the most common diagnosis with 47%, followed by Alzheimer's disease (28.9%) and dementia (19.1%). Almost 27% of diagnoses were due conditions like arteriosclerosis, senile dementia, and age‐related cognitive impairment.

**Conclusion:**

We noted a significant delay between cognitive symptoms onset and the initial consultation and also several incorrect diagnoses. The new disease‐modifying therapies has created an opportunity to improve AD subjects and family's life but it is imperative early and correct disease diagnosis. It is crucial to have population learning about the disease for early consultation and to improve medical knowledge for early diagnosis and treatment